# The Multidimensional Vaccine Hesitancy Scale: A Validation Study

**DOI:** 10.3390/vaccines10101755

**Published:** 2022-10-20

**Authors:** Beatrice Adriana Balgiu, Ruxandra Sfeatcu, Ana Maria Cristina Țâncu, Marina Imre, Ana Petre, Laura Tribus

**Affiliations:** 1 Department of Career and Educational Training, University Politehnica of Bucharest, 313 Splaiul Independenţei, 060042 Bucharest, Romania; 2 Department of Oral Health and Community Dentistry, Faculty of Dental Medicine, “Carol Davila” University of Medicine and Pharmacy, 17–21 Calea Plevnei Street, 010221 Bucharest, Romania; 3 Department of Complete Denture, Faculty of Dental Medicine, “Carol Davila” University of Medicine and Pharmacy, 17–21 Calea Plevnei Street, Sector 1, 010221 Bucharest, Romania; 4 Department of Aesthetics in Dental Medicine, Faculty of Dental Medicine, “Carol Davila” University of Medicine and Pharmacy, 17–21 Calea Plevnei Street, 010221 Bucharest, Romania; 5 Department of Internal Medicine, Faculty of Dental Medicine, “Carol Davila” University of Medicine and Pharmacy, 17–21 Calea Plevnei Street, 010221 Bucharest, Romania

**Keywords:** scale, vaccination hesitancy, validation

## Abstract

Vaccination hesitancy (VH) is a phenomenon which increases the occurrence of vaccine-preventable diseases. The study tests the validity of the Multidimensional Vaccine Hesitancy Scale (MVHS) in the case of a sample of Romanian adults (*n* = 528; Mean_age_ = 30.57). The latter filled in an online cross-sectional survey. The construct validity of MVHS was assessed by using confirmatory factor analysis (CFA), the reliability was calculated by using the internal consistency, and the convergent and discriminant validity was assessed by using the composite reliability (CR), and average variance extracted (AVE). The obtained model was invariant across gender. The structural equation model was designed for predictive validity by using the partial least square method (PLS-SEM) which analyses the relation between the MVHS dimensions and the vaccination willingness. The results show support for the 8-factor structure of the scale (χ^2^/df = 2.48; CFI = 0.95; RMSEA = 0.053). The Cronbach’s coefficients α > 0.70; McDonald’s ω > 0.70 and CR > 0.80 have very good values. The structural equation model shows that there are more dimensions of the scale which predict vaccination hesitancy in various types of vaccines—the main predictors remain the dimensions of health risk and healthy condition. The study’s conclusion led to the idea that the MVHS is suitable for medical practice and for research on the analysis of vaccination behaviours and intentions.

## 1. Introduction

Vaccination hesitancy (VH) is defined as “the delay to accept or the refusal to get vaccinated, despite vaccination services being available” [1]. A more restrictive definition considers that VH refers precisely to “preoccupations regarding vaccines, irrespective of the actual vaccination” [2]. In Romania, the VH phenomenon was on the rise in the context of the SARS-CoV-2 pandemic, and it was visible in the case of various population categories [3,4,5,6]. The vaccination rate in East-European countries, such as Romania, was much lower than in the rest of the European Union, and it led to challenges and extreme pressure on the medical system [7,8,9,10,11]. The Romanian vaccination policy did not persuade many people to be vaccinated against COVID-19. Most of the population put up unjustified resistance against vaccination due to lack of minimal medical knowledge, disinterest in science, and rejection of scientific arguments, as well as media disinformation [7,12].

The medical scientific community constantly recommends examining the factors that foster the acceptance of vaccination and approaching each of them individually in order to encourage people to accept vaccines [13]. An instrument for VH assessment is important for epidemiological studies and for research, and it can help concretely identify the factors that lead to the refusal or the postponement of the vaccination. One of the instruments relevant to the measurement of attitudes regarding vaccination is a short, six-item scale called the VAX scale (Vaccination Attitudes Examination Scale [14]), the only one which was translated and used in Romanian literature [4,6]; other instruments that have been used are questionnaires devised by the authors upon examining the attitudes towards various types of vaccines [3,15,16].

Given this paucity of instruments in the Romanian language for the identification of the correlations and factors that lead to VH, we proposed the validation of the Romanian version of the Multidimensional Vaccine Hesitancy Scale (MVHS-RO).

The Multidimensional Vaccine Hesitancy Scale (MVHS) was developed and validated on American subjects [17] with the aim of assessing the multifaced vaccine hesitancy construct with more precision. The scale has eight factors, with four items each. Each factor of MVHS correlates significantly with measurement instruments for general vaccine perceptions (The Vaccine Hesitancy Scale [18]), vaccine confidence (The Vaccination Confidence Scale [19]), and vaccine acceptance (Vaccine Acceptance Scale [20]). The eight factors have good internal consistency—Cronbach alpha obtained high values in several studies in the case of the general adult population: between 0.85–0.92 [17], over 0.90 [21], and between 0.88–0.95 [22]. Both in exploratory factor analysis and confirmatory factor analysis, the factor loading for the 32 items of the scale is high, between 0.54 and 1.00 and 0.61 and 0.95, respectively [17].

The multidimensional model realised by Matt Howard [17] covers a large scope of the VH construct, and it creates concrete content by identifying eight dimensions of the latter: (1) health risks (e.g., vaccines can cause long-term health issues); (2) cost (e.g., vaccines cost too much); (3) physical pain (e.g., needles bother me when receiving a vaccine); (4) inconvenience (e.g., I am too busy to get a vaccine); (5) personal reactions (e.g., I have allergic reactions to most vaccines.); (6) access (e.g., vaccines are unavailable where I live.); (7) healthy condition (e.g., I do not need vaccines because I rarely get sick.); (8) forget (e.g., getting vaccines often slips my memory). The scale calculates a final score regarding the hesitancy behaviour of the vaccine by summing up the scores obtained from the eight scales.

## 2. Materials and Methods

### 2.1. Data Collection

The cross-sectional study was carried out on a convenience sample in order to allow a reasonable number of participants to fill in the questionnaire between February and June 2022 on the Google Forms platform. A survey in the Romanian language was posted on the online platform and shared on WhatsApp and Facebook. The respondents’ participation in the study was voluntary and free, the participants’ anonymity was ensured, and the study was conducted in accordance with the ethical standards on data collection in Romania. The survey was secured so that the questionnaires could be completed only once by the same person. The time needed to complete the questionnaire was around 6–7 min. The eligibility conditions were 18 years of age and Romanian citizenship and residency. The only criteria for the exclusion were underage and citizenship and residence outside Romania.

### 2.2. Translation and Adaptation of MVHS into Romanian

The adaptation of MVHS-RO went through the stages recommended by the WHO [23]. Initially, the agreement of the author of the MVHS, Matt Howard, was required and received. Then, the scale was translated from English into Romanian by a psychologist—one of the authors of the study—and a professional English translator. The synthesis of the two translations led to the Romanian version of the scale. The second stage consisted of the translation from Romanian into English made by two other translators, both advanced English speakers. The differences between the two translations were dealt with until a consensus was reached. The synthesis of this translation proved to be similar to the original version of the scale. One last stage consisted in piloting the instrument in the case of 21 master’s students over 18 years (Mean_age_ = 24.28), who assessed how easily the items could be read and understood. No major content modifications to the items were needed.

### 2.3. Measures

1. Multidimensional vaccine hesitancy scale–MVHS [17] measures vaccination hesitancy behaviour, and it has 32 items assessed on a scale from 1 (strongly disagree) to 7 (strongly agree).
2. The scale of COVID-19 vaccine willingness has two items assessed on a scale from 1 (extremely disinterested) to 7 (extremely willing): Please show how willing you are to get an anti-COVID-19 vaccine next year if (1) the vaccine is free and if (2) it costs 2500 lei (Romanian currency). The internal consistency coefficients are Cronbach’α = 0.71 (95%CI: 0.66–0.76) and McDonald’ s ω = 0.71 (95%CI: 0.67–0.75).
3. The scale of flu vaccine willingness. Similarly, two items were used on a scale from 1 (extremely disinterested) to 7(extremely willing): Please show how willing you are to get a flu vaccine next year if (1) it is free and if (2) it costs 2500 lei (Romanian currency). For the respective coefficients, the acceptable value, Cronbach’s α 0.67 (95%CI: 0.60–0.72) and McDonald’ s ω = 0.66 (95%CI: 0.60–0.70). The two scales were inspired by the studies of Howard [17], Perez et al. [24], and Shapiro et al. [25].

### 2.4. The Sociodemographic and Vaccination Data

The sociodemographic data collected within the sample took into consideration (1) age; (2) gender; (3) level of education; (4) the location of the domicile (urban/rural); (5) work sector (private/public); (6) geographical region; (7) flu vaccination over the last year; (8) anti-COVID-19 vaccination.

### 2.5. Statistical Strategies

The construct validity was tested by means of confirmatory factor analysis (CFA) using the maximum likelihood method of estimation. To this purpose, the coefficients χ^2^ (chi-square), df (degrees of freedom), χ^2^/df (criterion chi squared/df), CFI (comparative fit index), RMSEA (root mean squared error of approximation), SRMR (standardized root mean square residual), and IFI (incremental fit index) were used. The following common recommendations were taken into consideration: χ^2^/df has a good value if it is <3 [26]. CFI and IFI values are good when they are ≥0.95 [27]. RMSEA and SRMR have good values, almost 0.06 [28]. AIC coefficient (Akaike information criterion) is used to compare models: the model with lower values, namely the simpler one, is more desirable. For the model to fit the data, the χ^2^ test must be insignificant. However, a significant χ^2^ is not a reason to reject the model as this value is sensitive to the sample size [29]. The measurement of the gender invariance by means of multi group-CFA (MG-CFA) was based on the rules recommended by Rutkowschi and Svetina [30], so ΔCFI < 0.010 and ΔRMSEA < 0.010. The reliability was examined by means of α and ω coefficients considered acceptable when are over 0.70 and adequate when are over 0.80 [31]. The convergent validity was assessed using the average variance extracted (AVE) and the composite reliability (CR) with cutoff levels above 0.50 and 0.70, respectively [32]. The gender differences were calculated using the nonparametric Mann–Whitney U test. The relation between MVHS and the pro-vaccination attitude was analysed by using structural equation modelling (PLS-SEM). In this case, the reliability was assessed by Dijkstra–Henseler’s rho (ρA), Jöreskog’s rho (ρc), and Cronbach α coefficients. It is recommended that these values be >0.70 [33]. The convergent and discriminant validity was verified by AVE and heterotrait monotrait ratio of correlations (HTMT), whose recommended cut-off levels are >0.50 and <0.85, respectively [33,34]. Multicollinearity was calculated by means of the variance inflation factor (VIF), whose value must not be >5 [35]. For all the analyses, SPSS v22, Amos v22 (IBM, New York, NY, USA), JASP 0.16.1 (University of Amsterdam, Amsterdam, The Netherlands), and ADANCO 2.3.1 [33] were used.

## 3. Results

### 3.1. The Sociodemographic Analysis of the Sample

The sociodemographic composition of the sample (*n* = 528; Mean_age_ = 30.57; S.D. = 14.40) shows the relatively equal proportion of the subsamples of male respondents (*n* = 215; 40,70%; Mean_age_ = 30.13; S.D. = 15.09) and female respondents (*n* = 313; 59.30%, with the Mean_age_ = 30.88; S.D. = 13.93) (Table 1).

The criterion “residence” shows that 83.7% come from the city and that the remaining 16.30% live in the country. In the sample, there is a higher number of subjects with university studies (61.60%) and post-university studies (25.00%). Regarding the work sector, 42.00% of the respondents work in the public sector, 46.27% in the private sector, 9.46% are freelance, and 2.27% are unemployed. The geographical distribution emphasize an overrepresentation of the respondents who live in the capital (55.30%), followed by participants from southeastern Romania (29.20%). A small number of people come from the northwest of Romania (3.20%).

### 3.2. Vaccination Data

In terms of vaccination, 374 people (70.80%) did not have their flu vaccine over the last year, while only 146 participants (27.70%) were vaccinated against flu over the last year. In total, 101 respondents (19.1%) were not vaccinated against COVID-19, while 419 subjects (79.40%) had the COVID-19 vaccine.

### 3.3. The Factorial Structure

For the analysis of the factorial structure, the 8-factor, 32-item model proposed by Howard [17] was tested. In order to mitigate the violation of multivariate normality (the shown Mardia coefficient had the excessive value of 801.750 and the critical ratio—c.r. = 203.259) the Bollen–Stine boostrapping with 5000 resamplings at bias-corrected confidence level in order to solve non-normality. The result led to a first model: χ^2^ = 1256.309; df = 436; χ^2^/df = 2.88; CFI = 0.93; IFI = 0.93; RMSEA = 0.060 (90%CI—0.056–0.064); SRMR = 0.061; AIC = 1504.301; *p* < 0.001. Although the values of the coefficients χ^2^/df, RMSEA and SRMR are good, the values of CFI and IFI are still low. The examination of the standardized factor loading revealed low loading for item 11 (getting a vaccine hurts), namely, 0.30. The elimination of the item led to a new model in which the main gain is the improvement of the values of the coefficients CFI and IFI: χ^2^ = 1122.739; df = 406; χ^2^/df = 2.76; CFI = 0.94; IFI = 0.94; RMSEA = 0.058 (90%CI—0.054–0.062); SRMR = 0.053; AIC = 1364.739; *p* < 0.001. The examination of the modification indices (MIs) led to the correlation of the errors of six items, two within Factor 1 (items 1 and 2 and 1 and 3), two within Factor 4 (items 13 and 14 and 13–16), and two within Factor 7 (items 27–28 and 25–26). Thus, the new result consisted of the improvement of the CFI and IFI coefficients, and AIC is apparently lower than the value of the other models which is an advantage for the new model [36]: χ^2^ = 994.810; df = 400; χ^2^/df = 2.48; CFI = 0.95; IFI = 0.95; RMSEA = 0.053 (90%CI—0.049–0.057); SRMR = 0.051; AIC = 1248.810; *p* < 0.001 (Table 2).

The significant value of χ^2^/df can be due to the large size of the sample, and it cannot be considered a criterion for the rejection of the model [31]. The standardized factor loading in the case of all the 31 items is >0.50, and therefore, the latter can be retained, following the recommendations of Pituch and Stevens [37]. The lower factor loading is displayed in the case of items 32 and 20 (0.51 and 0.58, respectively); for the others, there are five items between 0.60–0.70, two items between 0.70–0.80, and 22 items over 0.80 (Table 3). Model 3 was retained in the subsequent analyses.

### 3.4. Gender Invariance

The results obtained in gender invariance (Table 4) demonstrated that the scale measures the same construct for both gender groups, the modifications of coefficients CFA and RMSEA are smaller than the limit recommended in all three cases [30]. Although CFI did not reach the heuristic limit values, other fit coefficients suggest a reasonable fit of data (ΔCFI and ΔRMSEA). Therefore, we reached the conclusion that the invariance was accepted.

### 3.5. The Descriptive Analysis of the Data Obtained

As Table 5 shows, the highest values were obtained for the dimension-perceived risks (M = 11.50; S.D. = 6.14), which underlines the fact that those perceptions are valued in the case of vaccination and the fact that the respondents fear the possible risks related to vaccination. At the other end of the spectrum, the dimension access has the lowest average (M = 5.32; S.D. = 2.77). Related to gender, a single difference was obtained in the case of the “healthy” subscale (Mean_males_ = 9.19; S.D. = 6.62; Mean_females_ = 7.34; S.D. = 5.20; Mann–Whitney U = 39,114.00; *p* < 0.001) shows higher scores for the subsample of male respondents in comparison to female respondents.

Regarding the association of the data with the age of the respondents, we resorted to cross-tabulation between age groups: 18–29 years, 30–49 and 50–76 (Table 6) and vaccination hesitancy grouped according to the mean and standard deviation in three large groups: low-, moderate-, and high-level. The value of χ^2^ (4) = 6.236; df = 4; *p* = 0.188 shows that there is no statistically significant interdependence between the two analysed variables.

For reliability, α and ω coefficients for the total score of MVHS-RO have equal values of 0.93 (95%CI—0.92–0.94) (Cronbach’s alpha if item was deleted shows values between 0.92 and 0.93). All eight subscales have a high consistency (α and ω over 0.80), except for subscale access, in the case of which an acceptable coefficient is obtained: α = 0.71 and ω = 0.72 (Table 5). The elimination of item 11 led to higher reliability of the scale Pain, an increase from 0.80 to 0.90.

### 3.6. The Convergent and Discriminant Validity

The average variance extracted (AVE) and composite reliability (CR) were measured on the basis of factor loading (λ) and standard error of measurement (ε) obtained in CFA. As Table 7 shows, the AVE values for each subscale are over the minim cutoff level of 0.50, except for subscale access, for which AVE is 0.41. However, an AVE value <0.5 can be accepted if CR > 0.6 since the convergent validity of the construct is still appropriate [38]. In the case of CR, all values are between 0.73 and 0.94 and are therefore above the cutoff level of 0.70 [32]. For the discriminant validity of the scale, the Fornell–Larcker criterion was used; this criterion is about the comparison of the AVE square root from every construct to its interconstruct correlation.

All the correlations between MVHS subscales are significant at *p* < 0.001. Since the AVE square root is higher than the interconstruct correlations, the discriminant validity is confirmed. Upon examining Table 7, one can state that MVHS meets the discriminant validity criterion. The intercorrelations between the MVHS dimensions are between 0.17 and 0.57.

### 3.7. Predictive Validity

In order to analyse the MVHS prediction regarding the attitude towards the anti-COVID-19 and flu vaccination willingness, two SEM-PLS models were devised; the dependent variables were (1) COVID-19 vaccine willingness and (2) flu vaccine willingness. To this purpose, the bootstrap procedure was applied according to a recommendation by Henseler [34] of 5000 resamplings (95% confidence interval). Firstly, the relationship between MVHS and the anti-COVID-19 vaccine willingness was analysed. All the indicators having to do with the characteristics of the model are high: Dijkstra–Henseler’s rho (ρA), Jöreskog’s rho (ρc) (CR) are >0.80, and α Cronbach >0.70; the AVE values for the convergent validity are >0.50; HTMT < 0.85 highlights sufficient discriminant validity. The highest HTMT value is 0.64; the multicollinearity coefficients (VIF) are between 1.34 and 4.49 and are therefore <5 [35]. In order for the correlations of the latent variables to be considered significant, the value of t > 1.96 and the levels of the value of *p* < 0.05 [32].

**Figure 1:**
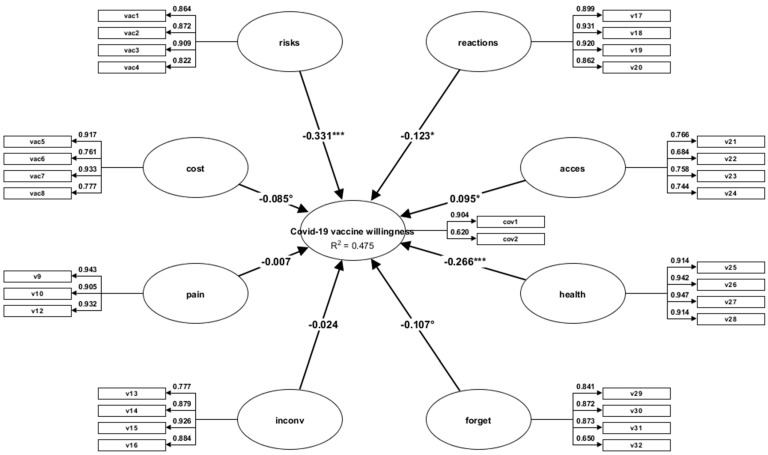
Variance-based structural equation modelling representing the MVHS prediction over anti-COVID-19 vaccine willingness; note: The figure shows the path (β) coefficients, which highlight the links between the structural model variables; v1−v32 = MVHS items. * *p* < 0.05; *** *p* < 0.001.

Table 8 shows the data of the structural model: independent variables Risks (β = −033; t = −5.74; *p* = 0.000; Reactions (β = −0.12; t = −1.99; *p* = 0.045), Access (β = −0,09; t = 2.07; *p* = 0.038) and Healthy (good personal health condition) (β = −0.26; t = −4.98; *p* = 0.000) predict anti-COVID-19 vaccine willingness. The MVHS dimensions account for 47% of the variance of anti-COVID-19 vaccine willingness (Figure 1).

In order to analyse the relationship between the MVHS dimension and the flu vaccine willingness, the characteristics of the model were calculated in the same way. The values of the coefficients which measure reliability, and the convergent and discriminant validity are good. Only in the case of the flu vaccine willingness variable, Cronbach’s α has an acceptable value of 0.67. VIF scores are less than 5.

**Figure 2:**
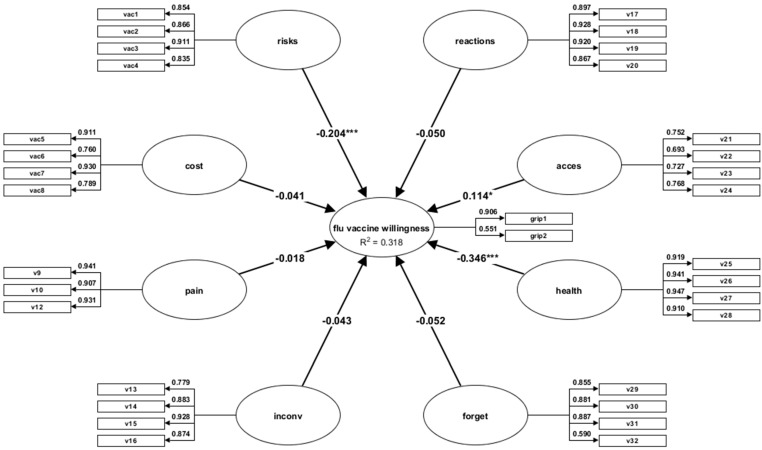
Variance-based structural equation modelling representing the MVHS prediction over flu vaccine willingness; note: The figure shows the path (β) coefficients, which highlight the links between the structural model variables; v1−v32 = MVHS items. * *p* < 0.05; *** *p* < 0.001.

The examination of the path coefficients (Table 9) shows that the flu vaccine willingness is predicted by Health risks (β = −0,20; t = −3.36; *p* = 0.000), Access (β = −0.11; t = −2.22; *p* = 0.026), and Health condition (β = −0.34; t = −6.07; *p* = 0.000). As Figure 2 shows, the MVHS dimensions account for 31% of the variance of the variable flu vaccine willingness.

Unlike for anti-COVID-19 vaccine willingness, in this case, the variance shows a relatively small effect. However, the variance is interpreted in the context of the study and the complexity of the model, which includes eight dimensions of the MVHS and a sample of over 500 people. It is possible that there is another fit of the model on another sample drawn from the same population. It is very possible that the reduced value of the variance is also the result of the flu vaccine willingness construct, which is measured only by two items—one of which, as seen in Figure 2, has a factor loading of 0.55.

An overview shows that the MVHS dimensions represent predicting factors for the anti-COVID-19 and flu vaccine willingness. Thus, one can conclude that the MVHS’s predictive validity was confirmed.

## 4. Discussion

The present study is the first to test the psychometric properties of MVHS in a culture which differs from the one in which the scale was developed—namely the East-European one, more precisely on the Romanian adult general population. The data analysis confirms that MVHS-RO has eight factors; item 11 was eliminated from the 32 items because the factor loading is very low (0.31). The gender invariance was supported for the Romanian version, which suggests that men and women have similar interpretations regarding multidimensional vaccine hesitancy. The convergent and discriminant validity of the scale was supported by the values obtained by AVE, CR, and the Fornell–Larcker criterion. The internal consistency for all the subscales demonstrated by means of Cronbach alpha and McDonald omega is high—over 0.80—except for the subscale Access, which has coefficients at an acceptable level of over 0.70. One gender difference for good health condition (healthy subscale) shows men perception regarding the good health and the immunity of their own bodies. The result suggests that men are more confident about their health and that that is why they consider vaccination unnecessary. The male subjects believe that their health is good enough, so they do not think they need to be vaccinated.

For the relation between MVHS and the positive attitude towards the anti-COVID and flu vaccine, the model of structural equation revealed that the dimensions of health risks, reactions, access, and healthy predict anti-COVID-19 vaccine willingness, while flu vaccine willingness is predicted by the dimensions health risks, access, and healthy, thus highlighting the importance of a set of dimensions for VH. The variance that the MVHS dimensions quantify in the willingness to vaccinate is still small. However, we consider the result to suggest that several MVHS dimensions are important for VH. The findings suggest that more MVHS dimensions are essential in understanding the intentions and behaviours related to vaccination. Just like in the case of the study in which MVHS was developed [17], in the present study, every dimension of VH had negative relations with vaccine willingness, except for the dimension access, which contains the perception of the fact that vaccines are difficult to receive. In the case of flu vaccine willingness, those who reported reduced access are more willing to get the flu vaccine (β = 0.08; t-value = 2.05).

Given that PLS-SEM is a causal modelling approach [35], one can state that the perception of health risks, the belief that vaccines can cause diseases, and the good perception of one’s health condition are the main causes of VH. The result corroborates other research findings. For example, scientific literature shows that the perception of risk is the most extended cause of vaccination hesitancy. In a meta-analysis, Brewer et al. [39] demonstrated that risk perception is a predictor of adults’ vaccination behaviour. In COVID-19 vaccination hesitancy, the meta-analytic study of Troiano and Nardi [40] showed that the most frequent reasons for which they refused to get vaccinated were the following: concerns related to safety/the fact that a vaccine created too quickly is too dangerous. Similarly, Soares et al. [41] identified the factors associated with refusal to and delay in becoming vaccinated, such as distrust of COVID-19 and health services available during the pandemic. The lack of trust in the anti-COVID-19 vaccine seems to be a universally valid cause as long as it is found in various cultural spaces, such as the rural population from South Africa [42]. The multidimensionality of VH is a fact that leads to the need for various strategies in understanding vaccination refusal. As the investigations carried out on representative cohorts show, it is required that public health systems follow the need for multifaceted strategies adapted to the needs and perspectives of undervaccinated people [43,44].

We admit to several limits of the study. The convenience sample is not representative of the general population, which restricts the generalisation of the results. Additionally, the cross-sectional design of the study does not allow the examination of the dynamic aspect of the scale. Future longitudinal studies should be carried out. Other limitations stem from the disparity in age, residence, and education of the sample participants that made it difficult to associate VH with other demographics. Lastly, there is a need to reconsider the method of measuring vaccination willingness as a multi-item construct.

Taking into consideration the results we obtained, one can conclude that MVHS-RO is a robust, psychometrically sound measure for VH. It can be used by researchers and the medical community interested in the divisive issue of vaccines, and it can help us become informed in case of the development of interventions whose role is to understand the nature of vaccine hesitancy.

## Figures and Tables

**Table 1:** The sociodemographic characteristics of the analysed sample.

Variables		*n* = 528
Age	18–76 years	Mean age: 30.57 years;
Gender	Males Females	40.70% 59.30%
Residence	Urban Rural	83.70% 16.30%
Education	Secondary studies University studies Post-university studies	13.40% 61.60% 25.00%
Work sector	Public Private Freelance Unemployed	42.00% 46.27% 9.46% 2.27%
Geographic region	Capital South-East South-West North-West	55.30% 29.20% 12.30% 3.20%

**Table 2:** Goodness-of-fit indices of CFAs.

Models	χ^2^	df	χ^2^/df	CFI	IFI	RMSEA (90%CI)	SRMR	AIC
M1	1256.309	436	2.88	0.93	0.93	0.060 [0.056–0.064]	0.061	1504.309
M2	1122.739	406	2.76	0.94	0.94	0.058 [0.054–0.062]	0.053	1364.739
M3	994.810	400	2.48	0.95	0.95	0.053 [0.049–0.057]	0.051	1248.810

M1-8 factors; M2-8 factors without item 11; M3-8 factors without item 11 and 6 correlated errors.

**Table 3:** Confirmatory factor analysis item loadings, mean and standard deviation of the items.

Items	Factor Loading	M	S.D.
1. Vaccines can cause long-term health issues.	0.81	2.84	1.85
2. Vaccines are unsafe.	0.86	2.53	1.66
3. Vaccines can cause illness.	0.86	2.65	1.71
4. Vaccines can cause certain disorders.	0.73	3.45	1.85
5. Vaccines cost too much.	0.88	2.33	1.64
6. I am unable to get vaccines because they cost too much.	0.68	1.66	1.26
7.Vaccines are too expensive.	0.94	2.17	1.60
8. Without health insurance, vaccines cost too much.	0.68	2.66	1.85
9. Needles bother me when receiving a vaccine.	0.95	2.23	1.89
10. I worry about needles when getting a vaccine.	0.80	2.20	1.85
11. Getting a vaccine hurts.	0.30	4.24	1.69
12. I have a phobia of needles when receiving a vaccine.	0.92	2.18	1.90
13. I am too busy to get a vaccine.	0.66	1.63	1.16
14. Getting a vaccine is too much of a hassle.	0.87	1.77	1.33
15. Getting a vaccine is too much trouble.	0.88	1.76	1.35
16. I do not have the time to get a vaccine.	0.77	1.53	1.09
17. I have allergic reactions to most vaccines.	0.85	1.78	1.37
18. I am a high-risk person for having a negative reaction to vaccines.	0.92	1.69	1.42
19. I am allergic to certain ingredients in vaccines.	0.89	1.77	1.46
20. I have a medical condition that prevents me from getting vaccines.	0.80	1.63	1.38
21. Vaccines are unavailable where I live.	0.58	1.30	0.98
22. There is nowhere to get a vaccine.	0.62	1.25	0.81
23. It is difficult to get a vaccine where I live.	0.71	1.26	0.82
24. It is difficult to know where to get a vaccine.	0.64	1.50	1.11
25. I do not need vaccines because I rarely get sick.	0.87	2.20	1.70
26. My strong immune system eliminates any need for vaccines.	0.91	2.10	1.65
27. I do not need vaccines because I am a low-risk person.	0.93	2.01	1.54
28. People in my physical condition do not need vaccines	0.87	1.77	1.43
29. Getting vaccines often slips my memory.	0.81	1.90	1.45
30. I just forget about getting vaccines.	0.85	1.73	1.47
31. I just never get around to getting vaccines.	0.80	2.02	1.66
32. I accidentally skip getting vaccines.	0.51	1.49	1.10

Note: In the second column—standardized factor loadings; M—mean, S.D.—standard deviation.

**Table 4:** Measurement invariance for MVHS-RO.

Model Invariance	Overall Fit Indices					Comparative Fit Indices			
	χ^2^	df	χ^2^/df	CFI	RMSEA	Δχ^2^	Δdf	ΔCFI	ΔRMSEA
Configural	1699.449	800	2.12	0.928	0.046	–	–	–	–
Metric	1787.958	823	2.17	0.922	0.047	88.509	23	0.006	−0.001
Scalar	1843.737	854	2.15	0.920	0.047	144.288	54	0.002	0.000
Strict	1941.946	890	2.18	0.915	0.047	242.497	90	0.005	0.000

**Table 5:** The descriptive analysis (mean, S.D., skewness, kurtosis, consistency coefficients).

Variables	M	S.D.	Min-Max	Skewness	Kurtosis	α	ω
Risks	11.50	6.14	4–28	0.77	−0.14	0.88	0.89
Costs	8.84	5.43	4–28	1.21	0.86	0.86	0.87
Pain	6.63	5.23	3–21	1.42	0.85	0.91	0.92
Inconvenience	6.70	4.30	4–26	1.94	3.55	0.89	0.89
Reactions	6.88	5.10	4–28	2.16	4.20	0.95	0.92
Access	5.32	2.77	4–22	2.80	8.77	0.71	0.72
Healthy	8.09	5.88	4–28	1.72	2.34	0.94	0.94
Forget	7.15	4.68	4–25	1.76	2.63	0.83	0.85
Vaccine hesitancy	65.39	27.50	31–155	1.10	0.55	0.93	0.93

**Table 6:** The relationship between age and vaccine hesitancy total score.

	Age Group (Years)		
Vaccine Hesitancy (%)	18–29	30–49	>50
low	11.90	11.50	61.30
moderate	74.40	69.50	61.30
high	13.80	19.11	22.70

**Table 7:** Intercorrelations and convergent and discriminant validity.

Factors	CR	AVE	1	2	3	4	5	6	7	8
Risks	0.88	0.66	**0.81** *							
Costs	0.87	0.64	0.35	**0.80** *						
Pain	0.92	0.79	0.24	0.20	**0.88** *					
Inconvenience	0.87	0.64	0.44	0.39	0.34	**0.80** *				
Reactions	0.92	0.75	0.52	0.26	0.24	0.57	**0.86** *			
Access	0.73	0.41	0.25	0.32	0.17	0.48	0.36	**0.64** *		
Healthy	0.94	0.80	0.54	0.27	0.23	0.57	0.51	0.33	**0.89** *	
Forget	0.83	0.57	0.35	0.29	0.25	0.62	0.36	0.49	0.58	**0.75** *

Note: * Square root of AVE value for each factor.

**Table 8:** Effects inference—anti-COVID-19 vaccine willingness.

Constructs	β	SE	t-Value	*p*-Value
Risks→COVID-19 vaccine	−0.33	0.05	−5.74	0.000
Costs→COVID-19 vaccine	−0.08	0.04	1.81	0.069
Pain→COVID-19 vaccine	−0.00	0.04	−0.14	0.882
Inconvenience→COVID-19 vaccine	−0.02	0.06	−0.36	0.716
Reactions→COVID-19 vaccine	−0.12	0.06	−1.99	0.045
Access→COVID-19 vaccine	0.09	0.04	2.07	0.038
Healthy→COVID-19 vaccine	−0.26	0.05	−4.98	0.000
Forget→COVID-19 vaccine	−0.10	0.05	−1.94	0.053

**Table 9:** Effects inference—flu vaccine willingness.

Constructs	β	SE	t-Value	*p*-Value
Risks→flu vaccine	−0.20	0.06	−3.36	0.000
Costs→flu vaccine	−0.04	0.05	−0.77	0.435
Pain→flu vaccine	−0.01	0.04	−0.35	0.721
Inconvenience→flu vaccine	−0.04	0.06	−0.62	0.531
Reactions→flu vaccine	−0.04	0.06	−0.75	0.48
Access→flu vaccine	0.11	0.04	2.22	0.026
Healthy→flu vaccine	−0.34	0.05	−6.07	0.000
Forget→flu vaccine	−0.05	0.05	−0.88	0.377

## Data Availability

The data presented in this study are available from the corresponding authors upon reasonable request.
